# Pleistocene archaeology and environments of the Free State, South Africa

**DOI:** 10.1080/0067270X.2024.2379724

**Published:** 2024-08-02

**Authors:** Michael B. Toffolo

**Affiliations:** Geochronology and Geology Programme, Spanish National Research Centre for Human Evolution (CENIEH), Paseo Sierra de Atapuerca 3, 09002 Burgos, Spain

**Keywords:** Pleistocene, South Africa, Free State, grassland, palaeoenvironments, Stone Age

## Abstract

Pleistocene climate variability is often seen as a major cause of much of the evidence observed in the archaeological and palaeontological record of Africa. While continent-wide climate systems play an important role when testing pan-African human evolutionary processes, a more focused perspective centred on specific ecosystems at a regional level allows a detailed assessment of the different spatiotemporal scales of the proxies used to reconstruct past environments and the ways humans adapted to their change over time. Recent research in the arid interior of South Africa has provided insights into the availability of freshwater in the open landscape, which is a fundamental factor for human survival and the spatiotemporal distribution of which may have had a major influence on adaptive strategies. This article reviews the Pleistocene archaeological and environmental evidence of the Free State province of South Africa, which has produced major localities such as Cornelia-Uitzoek, Florisbad and Rose Cottage Cave, with the aim of providing a starting point for the discussion over freshwater availability with regard to southern Africa’s Grassland Biome. Particular emphasis is given to the description of multi-proxy approaches including the analysis of sediments, faunal remains, enamel stable isotopes, pollens and phytoliths and absolute dating based on trapped-charge methods. The picture that emerges highlights the paucity of Pleistocene datasets in the Free State and the necessity to expand research at open-air sites and improve the chronological resolution of human occupations and palaeoenvironmental proxies.

## RÉSUMÉ

La variabilité climatique du Pléistocène est souvent considérée comme une cause majeure d’une grande partie des données observées dans les archives archéologiques et paléontologiques de l’Afrique. Les systèmes climatiques à l’échelle du continent ont certainement un rôle important à jouer pour tester les processus évolutifs humains panafricains. Cependant, une perspective plus ciblée, centrée sur des écosystèmes spécifiques au niveau régional, permet l’évaluation détaillée des différentes échelles spatio-temporelles des proxys ou données indirectes utilisés pour reconstruire les environnements passés, et la manière dont les humains s’adaptèrent aux changements au fil du temps. Des recherches récentes menées dans l’intérieur aride de l’Afrique du Sud ont permis de mieux comprendre la disponibilité d’eau douce dans les paysages ouverts. Il s’agit d’un facteur fondamental pour la survie humaine, et dont la répartition spatio-temporelle peut avoir eu une influence majeure sur les stratégies d'adaptation. Le présent article passe en revue les données archéologiques et environnementales du Pléistocène de la province du Free State en Afrique du Sud, où figurent des localités importantes telles que Cornelia-Uitzoek, Florisbad et Rose Cottage Cave. Le but ici est de fournir un point de départ pour une discussion sur la disponibilité de l’eau douce dans le biome des prairies d’Afrique australe. Un accent particulier est mis sur la description d’approches multi-proxy, y compris l’analyse de sédiments, les restes fauniques, les isotopes stables de l’émail, les pollens et phytolithes, et les datations absolues basées sur des méthodes de charge piégée. L’image qui se dégage met en évidence la rareté des ensembles de données du Pléistocène dans le Free State, et montre la nécessité d’étendre les recherches sur les sites en plein air et d’améliorer la résolution chronologique des occupations humaines et des proxys paléoenvironnementaux.

## Introduction

Climate change has often been invoked as a causal factor in human evolution. In Africa the relationship between the two has been established mainly on archaeological and palaeoenvironmental datasets from the eastern portion of the continent, which features long stratigraphic sequences reaching into the Lower Pleistocene (e.g. deMenocal 2004; Kingston 2007; Potts 2013; Tierney *et al.* 2017; Potts *et al.* 2018, 2020; Foerster *et al.* 2022; Robakiewicz *et al.* 2023). However, in recent years southern Africa has produced land and ocean archives that may be linked to the development of human adaptive strategies and cultural traditions during the Middle and Upper Pleistocene (e.g. Bar-Matthews *et al.* 2010; Ziegler *et al.* 2013; Mackay *et al.* 2014; Burrough 2016; Carr *et al.* 2016; d’Errico *et al.* 2017; Caley *et al.* 2018; Ecker *et al.* 2018; Scott and Neumann 2018; Bamford 2021; Chase 2021; Hahn *et al.* 2021). Research in the South African interior has highlighted the importance of the Nama-Karoo, Succulent Karoo and Savanna Biomes for our understanding of the environmental context of human evolution (e.g. Lukich *et al.* 2020; Wilkins *et al.* 2021; Mackay *et al.* 2022; Carr *et al.* 2023; Phillips *et al.* 2023), although the different spatiotemporal scales inherent to different palaeoenvironmental proxies hinder a proper assessment of climate change at sub-continental scale (Lukich and Ecker 2022) and its purported causal relationship with milestones in human evolution (Faith *et al.* 2021).

As proposed by Butzer (1984) with regard to southern Africa as a whole, and more recently by Lukich and Ecker (2022) for the southern Kalahari, considering archaeological and palaeoenvironmental archives at a smaller geographic scale can help mitigate the discrepancies between different proxies and lead to more robust interpretations as to how local environments respond to large-scale climate mechanisms over time. Lukich and Ecker (2022) used evidence from multiple environmental proxies collected at sites in the Northern Cape Province of South Africa to determine the spatiotemporal distribution of freshwater and how that may have affected human groups in the last two million years in this portion of the Savanna Biome. Indeed, the availability of freshwater in the landscape may have had pervasive influence on local behaviours and the spatial distribution of human groups, since water is a fundamental factor for survival (e.g. Potts 2012). This becomes especially apparent in the interior of South Africa, which, starting from the Middle Pleistocene, was characterised by a general trend towards arid conditions (Ecker *et al.* 2018, 2020) including dry spells (Toffolo *et al.* 2017; Lukich *et al.* 2020), except for an interlinked system of lakes, springs and rivers that supported local animal populations and possibly human groups (Brink 2016).

**Figure 1.**
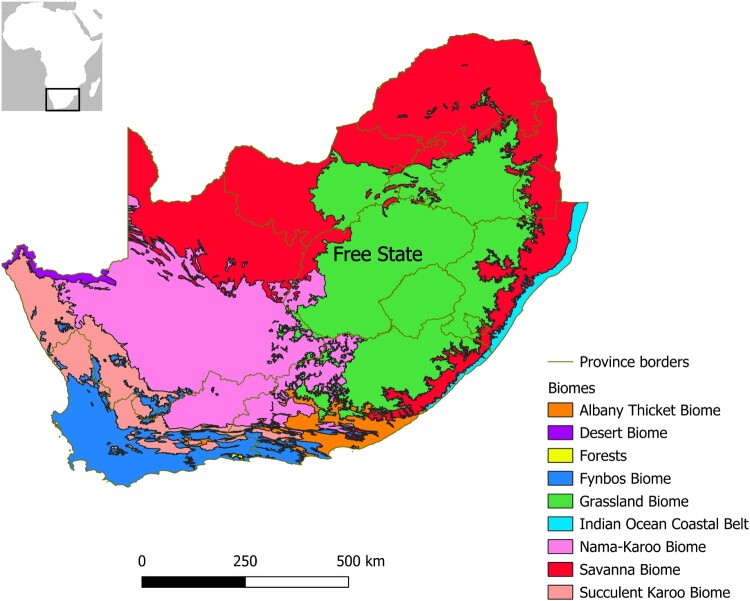
Biomes of South Africa, Lesotho and eSwatini, reproduced from Mucina and Rutherford (2006).

This article reviews the Pleistocene archaeological and environmental evidence of the Free State Province of South Africa with the aim of providing a basis for expanding the discussion over human presence relative to freshwater availability to the Grassland Biome, which in South Africa is mainly represented by the Free State (Figure 1). This geographically limited region, which also includes ecotones at the transition with the Nama-Karoo and Savanna Biomes, has produced a few major localities for the study of palaeoecology and human evolution, such as Cornelia-Uitzoek, Florisbad and Rose Cottage Cave (Wadley 1997; Kuman *et al.* 1999; Brink *et al.* 2012), some of which include, or are located next to, freshwater bodies in the open landscape. In addition, the position of the Free State, at the crossroads between the arid Kalahari basin, the well-watered Afromontane grasslands of the Maloti-Drakensberg mountain range and the Karoo semi-desert, makes it particularly important for a better understanding of human dispersal in the interior of the sub-continent in relation to local environmental change. Renewed research programmes at some of these localities, as well as at new sites, offer the opportunity to reconsider the available archaeological and palaeoenviromental evidence in the light of a multi-proxy approach and to identify current limitations and future research directions related to the investigation of the link between freshwater availability and human palaeoecology.

## Regional setting

The Free State is part of the inland plateau that occupies most of the interior of South Africa and is located at an elevation spanning between 1200 and 3000 m above sea level (a.s.l.). The portions of the province that exceed 1500 m a.s.l. are all located in its eastern part, where the Maloti-Drakensberg Mountains run in a northeast-southwest direction; the remainder, below 1500 m a.s.l., is characterised by rolling plains occasionally interrupted by isolated dolerite hills (‘kopjes’ in Afrikaans). The portion of the Free State between roughly 1300 and 2100 m a.s.l. is historically part of the Highveld, which includes also most of Gauteng and portions of the Eastern Cape, Northern Cape, North West, Limpopo and Mpumalanga provinces, as well as the lowlands of Lesotho.

The geology of the Free State is rather simple and mainly confined to the Karoo Supergroup, featuring Ecca Group shales (Permian) in the western plains and Beaufort and Stormberg Groups sandstones (Permian to Jurassic) in the eastern highlands (Loock and Grobler 1988; McCourt 2016). Within the Stormberg Group, the Clarens Formation is particularly important for a high density of caves and rock shelters that were occupied by hunter-gatherers during the Pleistocene and Holocene (Wadley 1995; Mitchell 2000). The highlands in the east are in some cases capped by Jurassic basalt, whereas the plains in the west are punctuated by Jurassic dolerite hills and sills that intruded the Ecca shales. The contact metamorphism between lava and shales produced the local knapping stone, called hornfels (also called ‘lydianite’ and ‘indurated shale’ in earlier literature), which was the main lithic raw material used by hunter-gatherers to manufacture stone tools (Sampson 1968, 1972; Kuman *et al.* 1999; Brink *et al.* 2012). In the eastern portion of the province, cryptocrystalline silicate rocks from the Maloti-Drakensberg were also used (e.g. Valladas *et al.* 2005). At the border with the North West Province, the Vredefort impact structure includes granites and gneiss (Reimold and Gibson 1996). The unconsolidated sediments on top of bedrock reflect the local geology, with fine sands rich in quartz, feldspars and magnetite as the dominant components of the coarse fraction. With regard to the fine fraction, kaolinites and smectites are the most common components, whereas carbonates are almost completely absent, except for pedogenic calcretes (Toffolo *et al.* 2017, 2019; Bousman *et al.* 2023a).

**Figure 2.**
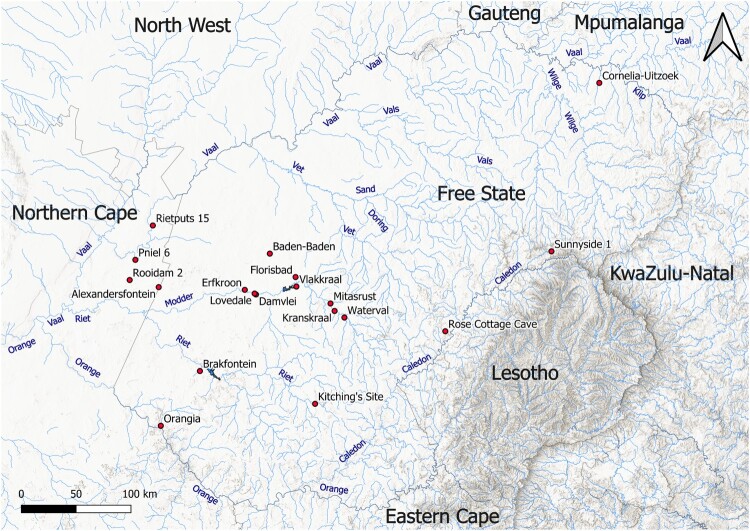
Topographic map of the Free State including hydrography, with the names of the main rivers and sites mentioned in the text. Source: Hydrography: Department of Water and Sanitation, South Africa. Topography: Esri. ‘Topographic’ [basemap]. ‘World Topographic Map’. October 26, 2017. https://www.arcgis.com/home/item.html?id=7dc6cea0b1764a1f9af2e679f642f0f5 (May 24, 2024).

The largest drainage systems are those of the Orange and Vaal Rivers, which flow westwards and represent the southern and northern borders of the Free State, respectively (Figure 2). Other major rivers include the Caledon at the border with Lesotho (flowing into the Orange to the south), the Klip and Wilge in the northeast, the Vals in the north and the Sand, Doring, Vet, Modder and Riet in the west (all flowing into the Vaal) (Tooth 2016). The confluence of the Vaal with the Orange and the land in between, which geographically belong to the drainage system of the Free State, are part of the Northern Cape Province as a consequence of the British annexation of Griqualand West in 1877, but are nevertheless considered here in relation to the occurrence of archaeological sites. Other river systems existed in the past, such as the palaeo-Kimberley, but these were disrupted by tectonic uplift during the Pliocene and thus became choked (Marshall 1988). As a result, endorheic basins formed, which over time dried up and transformed into deflation mudflats, or ‘pans’ (Marshall and Harmse 1992). These geomorphological features often occur as threads over long distances, which are reminiscent of the once active drainages (Grobler *et al.* 1988). Pans are concentrated in the western Free State and are almost always associated with a lunette dune in their southeastern margin, which is the result of sediment deflation by the prevailing northwest winds (Holmes *et al.* 2008; Rabumbulu and Holmes 2012; Holmes 2015). At different stages during the Pleistocene, these pans were actual lakes that supported large animal populations, whereas today they may fill up only during exceptional rainy seasons (Brink 2016).

Its inland location between two oceans and the elevation of the Free State are the main factors governing its climate, which is semi-arid in the west and temperate in the east, characterised by cold and dry winters and hot/warm and humid summers typical of southern Africa’s summer rainfall zone. Mean annual precipitation exhibits an east-west gradient reflecting the main source of rainfall from the Indian Ocean and reaches ∼730 mm in the northeast, whereas the western part is effectively semi-arid, receiving less than 500 mm and as little as 300 mm between the Riet and the Orange (Lynch 2004; Mucina and Rutherford 2006; Moeletsi and Walker 2012; Mbiriri *et al.* 2019). Summer rainfall often occurs as thunderstorms, which enhance surface erosion of sandy sediments by water runoff and promote the incision of ravines (‘dongas’ in Afrikaans) in river and lake terraces. Dongas are particularly important erosional features because they expose Pleistocene stratigraphy rich in archaeological and palaeontological localities (e.g. Bousman *et al.* 2023a).

The territory of the Free State falls almost completely within southern Africa’s Grassland Biome (Figure 1), more specifically the Mesic Highveld Grassland Bioregion in the east and the Dry Highveld Grassland Bioregion in the centre-west. The former is characterised mainly by chloridoid grasses, such as *Themeda triandra* and *Cymbopogon pospischilii*, and Karoo shrubs, which thrive especially on kopjes and in dongas. The latter is dominated by *Eragrostis curvula*, *Eragrostis capensis*, *Eragrostis plana* and a smaller proportion of *T. triandra*. The Eastern Kalahari Bushveld Bioregion of the Savanna Biome and the Upper Karoo Bioregion of the Nama-Karoo Biome are present in the western portion of the Free State, close to the border with North West and the Northern Cape. They comprise, respectively, mixed woodland-grassland dominated by *Acacia mellifera*, *Acacia tortilis* and *Acacia erioloba* and xeric shrubland dominated by dwarf Karoo shrubs and *A. mellifera* (Mucina and Rutherford 2006). These types of sparse plant cover favour erosional processes in dongas during the rainy season (October to March).

## Pleistocene archaeological and environmental evidence

**Figure 3.**
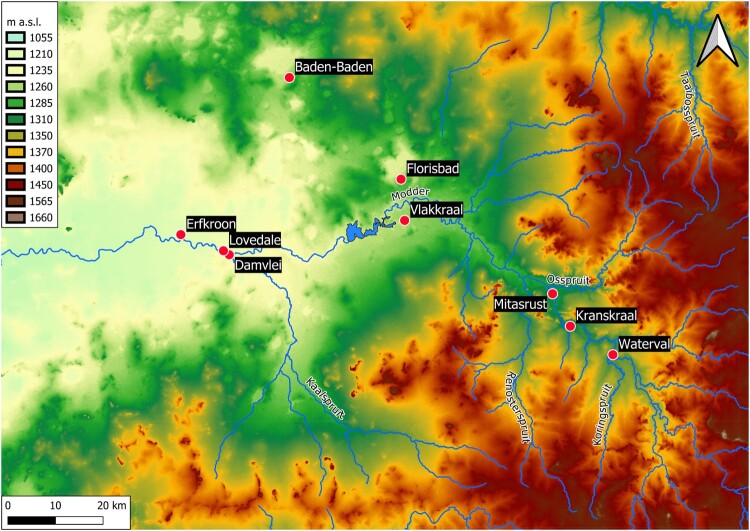
Map of the Modder River valley, showing the location of the sites mentioned in the text. Source: United States Geological Survey. Source: Shuttle Radar Topography Mission 1-arc second Global, National Geospatial-Intelligence Agency (NGA), USGS Earth Resources Observation and Science (EROS), retrieved from NASA Earthdata.

As in other parts of the interior of South Africa, archaeological research in the Free State started in the nineteenth century with the collection of surface artefacts and fossils in dongas and river terraces, especially in areas affected by diamond mining along the Vaal, which provided fresh exposures (Goodwin 1928; Cooke 1955). In following decades, surveys expanded to the Modder and Riet basins (Goodwin and van Riet Lowe 1929). Systematic excavations started only in the 1920s and focused mainly on open-air sites, since the sandstone geology conducive to rock-shelter formation is concentrated in the eastern highlands close to the Lesotho border. The Modder basin produced most of the early evidence, together with sporadic finds along minor rivers (e.g. van Hoepen 1930, 1932b; Dreyer 1938). Scientific excavation of rock-shelters started in the 1940s with Rose Cottage Cave and was later followed by other late Pleistocene and Holocene sites in the Caledon valley (Wadley 1995; Mitchell 2000). Except for the large-scale Orange River Scheme project in the south (Sampson 1968) and the continued excavations at Rose Cottage Cave (Wadley 1997), most of the research from the second half of the twentieth century onwards has taken place in the Modder basin, which also contributed to the establishment of the main palaeoenvironmental sequence in the Free State for the Middle and Upper Pleistocene (Figure 3).

### Lower Pleistocene

#### Cornelia-Uitzoek

**Figure 4.**
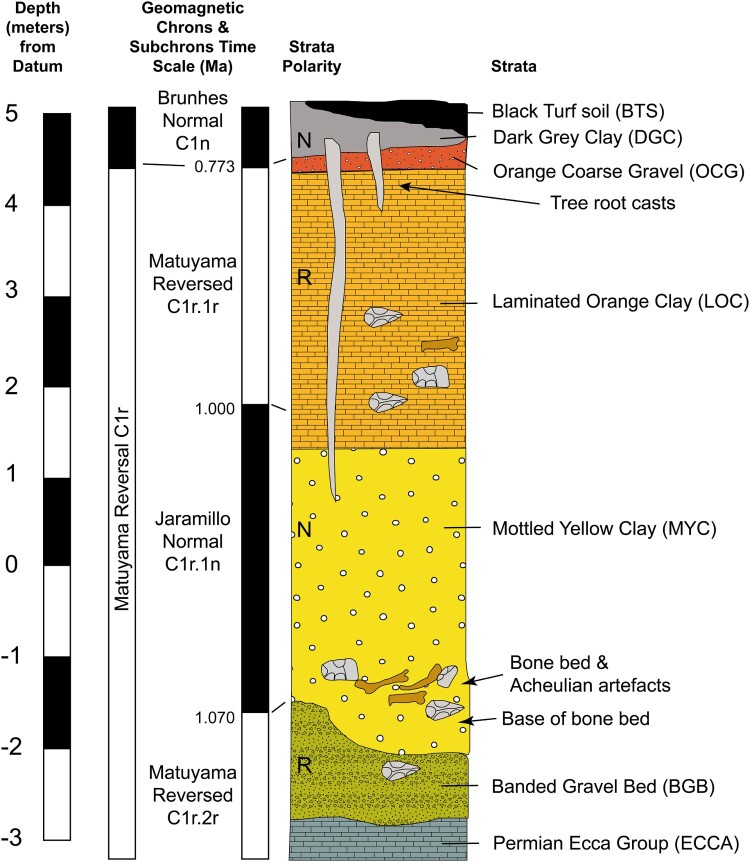
Stratigraphic sequence at Cornelia-Uitzoek, showing palaeomagnetic chronology and the position of artefacts and fossils. Reproduced from Bousman *et al.* (2023b: Figure 2) with permission from Springer Nature.

Sediments dated to the Lower Pleistocene (2.580–0.773 mya) are exceedingly rare in the Free State and so are archaeological and fossil localities. To date, the only known Lower Pleistocene site is Cornelia-Uitzoek in the northeast of the province (Brink *et al.* 2022; Bousman *et al.* 2023b). The site, located on the right bank of the Schoonspruit, a tributary of the Vaal, was first excavated by E.C.N. van Hoepen in the 1920s, who collected mammal fossils and artefacts (van Hoepen 1930, 1932a, 1932c, 1947). Further excavations were conducted in 1953 by A.C. Hoffman and A.W. Crompton, who found more fossils and Earlier Stone Age (ESA) and Middle Stone Age (MSA) artefacts (Butzer 1974; Clark 1974; Cooke 1974), and in the 1990s–2010s by J.S. Brink and colleagues, who recovered additional artefacts and fossils, including a *Homo* sp. molar (right M^1^), reconstructed the formation processes of the site and dated it to ∼1.0 mya using palaeomagnetism (Bender and Brink 1992; Brink and Rossouw 2000; Brink 2004; Brink *et al.* 2012; Toffolo *et al.* 2019) (Figure 4). A nearby locality, Cornelia-Mara, produced abundant sandstone artefacts in the lowermost sedimentary unit made of fluvial gravel; the artefacts are weathered by water transport (Butzer 1974).

The stratigraphic sequence at Cornelia-Uitzoek includes five sedimentary units formed through fluvial deposition under different flow regimes of the Schoonspruit in a channel cut into Ecca shale. From bottom to top, these are the Banded Gravel Bed (BGB), Mottled Yellow Clay (MYC), Laminated Orange Clay (LOC), Orange Coarse Gravel (OCG) and Dark Grey Clay-Black Turf Soil (DGC-BTS), all separated by unconformities (Figure 4). Acheulean artefacts (N = 154) and fossils were recovered primarily from the base of the MYC, although some assemblages were found at different elevations also within the LOC. The BGB produced a large assemblage of Acheulean stone tools and a few fossils, whereas MSA and LSA artefacts were found in the BTS, although away from the Acheulean contexts (Bousman *et al.* 2023b). The boundary between BGB and MYC corresponds to the transition between the Matuyama Reversed chron and the Jaramillo Normal chron, which is dated to 1.07 mya. The transition between the Jaramillo Normal and the Matuyama Reversed (1.0 mya) occurs in the lower portion of the LOC, while the following transition to the Bruhnes Normal chron (0.773 mya) corresponds to the boundary between OCG and BTS. All the sediment above this boundary dates to the Middle and Upper Pleistocene and Holocene. Immediately above the BGB-MYC boundary, Brink and colleagues identified a bone bed accumulated by hyaenas, as indicated by the presence of gnawing marks and hyaena coprolites, including Acheulean artefacts (Brink 2004; Brink *et al.* 2012). Micromorphological analysis showed that the bone bed accumulated on an exposed surface close to the river in a palaeo-donga system (Toffolo *et al.* 2019). The concentration of artefacts in the bone bed indicates a modest human occupation in the surroundings that was transported by colluvial processes into the bone bed within the palaeo-donga (Bousman *et al.* 2023b).

The most important contribution of Cornelia-Uitzoek to our knowledge of Lower Pleistocene environments certainly comes from the faunal remains, which represent the type assemblage of the Cornelian Land Mammal Age (LMA), transitional between the Makapanian (5.5–1.0 mya) and the Florisian (0.773–0.012 mya) (Hendey 1974b; Brink 2016; Van Couvering and Delson 2020). The Cornelian LMA features an earlier component showing a biogeographic connection with eastern Africa based on the presence of the genus *Kolpochoerus* and the species *Hippopotamus gorgops* and *Antidorcas recki* and of a derived component that marks the emergence of bovid populations adapted to open grasslands and wetlands in the semi-arid interior of South Africa, including southern endemics such as *Antidorcas bondi*, *Damaliscus niro* and *Connochaetes gnou laticornutus* (Brink *et al.* 2012, 2022; Bousman *et al.* 2023b). The occurrence of water-dependent and wetland-adapted species, such as *H. gorgops* and *A. bondi* (respectively), together with the sedimentary evidence for overbank deposition of the MYC and LOC, supports the interpretation that animal populations lived along a meandering river system within a large alluvial plain rich in wetlands and oxbows (Toffolo *et al.* 2019), similar to what can be observed today in the northeast of the Free State along the Klip River (Tooth *et al.* 2002). The analysis of carbon and oxygen stable isotopes of enamel from equids, suids, bovids and hippopotami revealed a mixed feeding signal in agreement with the mixed grassland-woodland habitats indicated by the faunal assemblage and consistent with the presence of abundant water sources (Codron *et al.* 2008; Brink 2016). The human molar exhibits traits consistent with early *Homo* and is the earliest human fossil known from South Africa outside the Cradle of Humankind (Brink *et al.* 2012).

#### Other localities

Other sedimentary pockets dated to the Lower Pleistocene are not known within the borders of the Free State. If one considers the portion of land between the confluence of the Orange and Vaal in the Northern Cape, fossils and Acheulean artefacts have been found on the left bank of the Vaal, mainly in surface scatters and within gravel beds (Rietputs Formation) and layers of windblown sand and alluvial silt (Riverton Formation) at Riverton, Nooitgedacht, Power’s Site (Pniel 1), Droogeveld, Rooipoort and Middelplaats North (Burkitt 1928; Goodwin 1928; Power 1955; Helgren 1977, 1978). Rietputs 15, dated to ∼1.3 mya by cosmogenic nuclides, yielded an assemblage of 1174 lithic artefacts of early Acheulean technology that is the precursor of the prepared core technology observed in the later Victoria West industry (Gibbon *et al.* 2009; Leader *et al.* 2018), an example of which is found on the opposite side of the river at Canteen Kopje (Li *et al.* 2017).

### Middle Pleistocene

#### Fauresmith sites

The first half of the Middle Pleistocene (0.773–0.129 mya) is *terra incognita* in the Free State as no archaeological or fossil localities are currently known except for the BTS sedimentary unit at Cornelia-Uitzoek, which is of Middle Pleistocene age or younger. The second half of the Middle Pleistocene saw the appearance of the Fauresmith lithic technocomplex in the interior of South Africa (∼470–230 kya), characterised by small handaxes associated with large blades and points based on Levallois technology (Lombard *et al.* 2022 and references therein). Possible Fauresmith artefacts have been collected from dongas and pans in the south and west of the Free State, mainly in the basins of the Orange, Riet and Modder, but they have never been dated due to the absence of primary depositional contexts (river gravels) or the sedimentary matrix necessary for trapped-charge dating (surface scatters). Known localities possibly related to the Fauresmith include the land between the junction of the Modder and Riet and along both sides of the Riet up to Koffiefontein (Rickard 1880; Goodwin and van Riet Lowe 1929), the area around Boshof (Johnson 1907; Goodwin and van Riet Lowe 1929), the areas around Fauresmith, Luckhoff, Edenburg and Philippolis (Goodwin and van Riet Lowe 1929) and the sites of Elandskloof 13 and Waterval 16B along the Orange (Sampson 1968). Sporadic surface finds of handaxes, including relatively small specimens, are mentioned also in recent contract archaeology reports about the area around Dealesville (e.g. Orton 2021). The only exception in terms of depositional context might be the type locality of the Fauresmith technocomplex, the farm Brakfontein, located between the towns of Koffiefontein and Fauresmith on a seasonal tributary of the Riet. The conditional is necessary because in this case the artefacts were found by C. van Riet Lowe in 1926 eroding out of the base of a sand dune, which may provide an intact sedimentary context suitable for absolute dating (van Riet Lowe 1927; Burkitt 1928; Goodwin and van Riet Lowe 1929; Underhill 2011, 2012). More recently, small handaxes consistent with the Fauresmith technocomplex have been found in dongas along the Sand and Doring, in the central-west Free State (de Ruiter *et al.* 2011).

Additional Fauresmith surface occurrences were found at the junction between the Orange and Vaal drainages in the Northern Cape (Goodwin 1928; Helgren 1978). At Rooidam 1, uranium-series dating of lacustrine calcrete yielded a minimum age of 174 ± 20 kya for the underlying Fauresmith assemblage (Szabo and Butzer 1979); at Rooidam 2, an average minimum age estimate of 174 ± 15 kya based on optically stimulated luminescence (OSL) dating of sediments was obtained for the Fauresmith assemblage (Eltzholtz 2020). Pniel 6, located near Power’s Site on the left bank of the Vaal, produced a surface assemblage of ESA lithics and three distinct collections of excavated lithics. The first two, derived from the excavations of P. Beaumont in the 1980s (Beaumont 1990) and Beaumont and J. McNabb in 2000, are only partially analysed and appear to be a palimpsest of different Acheulean and MSA assemblages mixed by colluvial processes (Underhill 2012). The excavations led by M. Ecker between 2017 and 2019 uncovered 48 faunal remains (unpublished) and 632 artefacts made mainly on hornfels from three excavations areas, which are consistent with the Fauresmith assemblages from Kathu Pan 1 Stratum 4a (Porat *et al.* 2010), but lack bifaces, choppers and polyhedrons (Ecker *et al.* 2021), in contrast with nearby sites such as Canteen Kopje (Kuman *et al.* 2020). Pniel 6 also produced surface scatters of Middle Pleistocene (Florisian) faunal assemblages dominated by equids and bovids and including water-dependent species such as *Kobus leche* and *Hippopotamus amphibius* (Hutson 2018).

#### Florisbad

The earliest dated Middle Pleistocene locality in the Free State is Florisbad (also known as Hagenstad in early literature), a spring site near Soutpan in the Modder basin (40 km northwest of Bloemfontein) that spans the last ∼300,000 years. Stone tools and fossils were found in 1912 during construction works of a bathhouse (Broom 1913; Collins 1915; Goodwin and van Riet Lowe 1929) and collections were expanded in following years (Dreyer and Lyle 1931). In 1932, T.F. Dreyer unearthed the partial cranium of a basal *H. sapiens* (then named *Homo helmei*) from an inactive spring vent in the lower levels of the site (Dreyer 1935, 1947; Drennan 1937; Galloway 1937; Rightmire 1978; Clarke 1985; Curnoe and Brink 2010; Smith *et al.* 2015; Bruner and Lombard 2020; Grün and Stringer 2023). During the 1930s excavations, Dreyer found a large number of faunal remains and MSA artefacts, including a wooden implement possibly consistent with a propulsor (Bamford and Henderson 2003), and established the stratigraphic sequence, which was organised around four major layers of peat (Dreyer 1938). Similar fauna and stone tools were found by Dreyer, and later by L.H. Wells, at the Vlakkraal (also known as Prinsloo) spring site, located 8 km south of Florisbad on the opposite side of the Modder, in today’s Soetdoring Nature Reserve (Dreyer 1938; Wells *et al.* 1942). Excavations at Florisbad in 1952–53 by A.C. Hoffman, A.J. Meiring and Dreyer produced more fauna and artefacts, especially from the basal layers (Meiring 1956). After being acquired in 1980 by the National Museum Bloemfontein, the site was again excavated by K. Kuman and R.J. Clarke in 1981–1984, whose work contributed to a better understanding of its archaeology, formation processes and palaeoenvironments (Rubidge and Brink 1985; Kuman and Clarke 1986; Brink 1987, 1988; Grobler and Loock 1988a, 1988b; Loock and Grobler 1988; Kuman 1989; van Zinderen Bakker 1989, 1995; Visser and Joubert 1990, 1991; Joubert *et al.* 1991; Joubert and Visser 1991; Kuman *et al.* 1999). The latest excavation campaign, led by Brink and Z. Henderson in the 1990s, produced more fossils and artefacts, new data on pollen and phytoliths and the first absolute dating of the sedimentary deposits and human fossil, which showed its Middle Pleistocene age (Brink and Lee-Thorp 1992; Scott and Brink 1992; Brink 1994; Brink *et al.* 1996; Grün *et al.* 1996; Brink and Henderson 2001; Henderson 2001; Scott and Nyakale 2002; Coetzee and Brink 2003; Scott and Rossouw 2005). In more recent years, research based on fossils from the collections and on sediments from renewed sections have produced more insights into palaeoenvironments and formation processes (Codron *et al.* 2008; Manegold and Brink 2011; Toffolo *et al.* 2015, 2017; Scott *et al.* 2019). Finally, a renewed luminescence dating programme confirmed the age of the Middle Pleistocene sequence and provided ages for the Upper Pleistocene and Holocene deposits (Pinder 2021).

**Figure 5.**
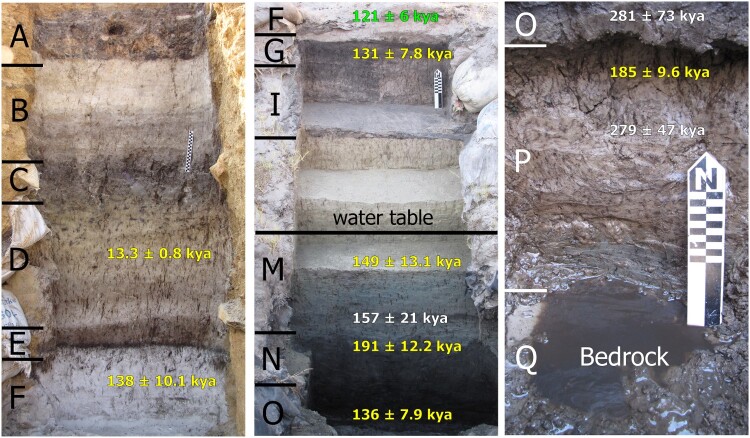
Stratigraphic sequence of the Dreyer section at Florisbad, from top (left) to bottom (right), including sedimentary unit according to Kuman *et al.* (1999), luminescence ages by Grün *et al.* (1996) in white and by Pinder (2021: Figure 7.1) in yellow, ESR age by Grün *et al.* (1996) in green and the level of the present-day water table. Scale bars: 20 cm.

**Figure 6.**
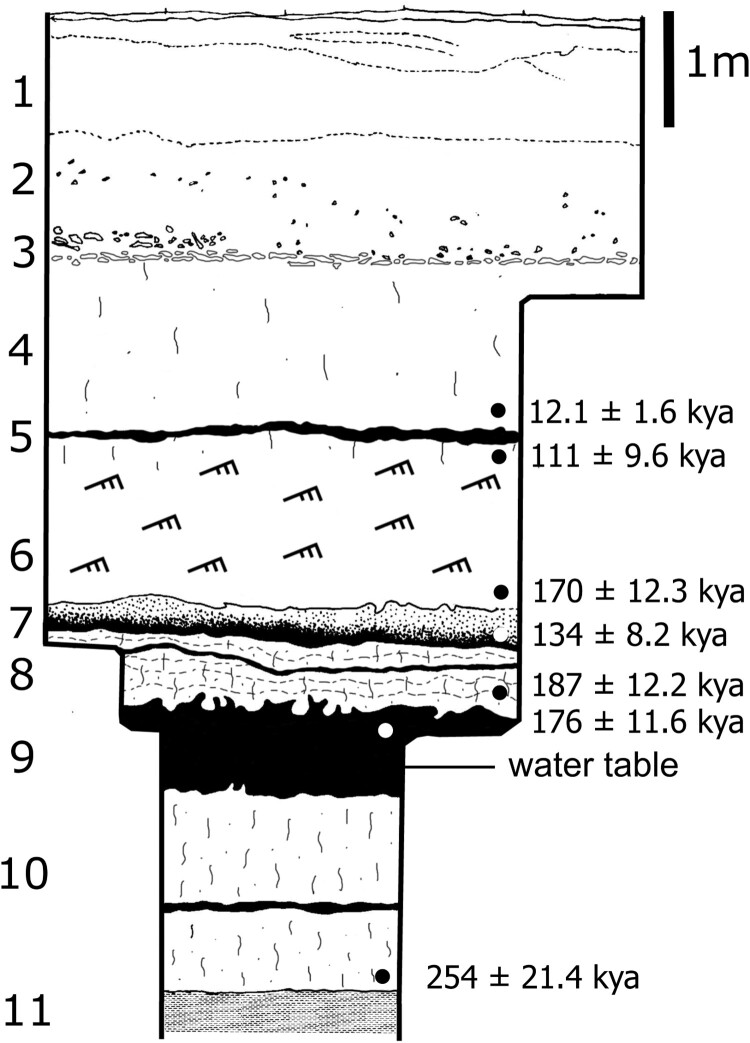
Stratigraphic sequence of Pit 3 at Florisbad, including unit numbers according to Toffolo *et al.* (2017), luminescence ages by Pinder (2021: Figure 7.1) mentioned in the text and the level of the present-day water table.

The Middle Pleistocene deposits at Florisbad include Units P to H of Kuman *et al.* (1999; Units 10 to 7 in Toffolo *et al.* 2017: Table 1) from bottom to top, which roughly represent the period from 300,000 to 120,000 years ago based on luminescence dating of quartz and feldspar and electron spin resonance (ESR) dating of tooth enamel (Grün *et al.* 1996; Pinder 2021) (Figures 5 and 6; Table 1). These sedimentary units comprise mainly fine sand and silt and were accumulated through aeolian deposition and reworked by spring water (P), which favoured the formation of peat under waterlogged conditions (O–N, L–H) and a shallow pool close to the present-day spring eye (M) (Toffolo *et al.* 2017). The stratigraphic sequence is in places disrupted by the extinct spring vents, now filled with sand, that once issued water to the surface and thus ‘captured’ the bones that happened to be in the vicinity. The latter eventually fossilised due to the favourable chemistry of the spring water (Toffolo *et al.* 2015 and references therein). The great number of faunal remains from the vents contributed to the definition of the Florisian LMA, which was characterised by open grasslands rich in large- to small-sized ungulates in a significantly wetter environment compared to the Holocene, including extinct species such as *A. bondi*, *D. niro*, *Equus capensis*, *Megalotragus priscus*, *Syncerus antiquus* and possibly *Equus lylei* (Hendey 1974a 1974b; Klein 1984; Brink 1987, 1988, 1994, 2016; Brink and Lee-Thorp 1992; Lacruz *et al.* 2002; Van Couvering and Delson 2020). The wetland character of the Florisian grasslands is apparent based on the occurrence of water-dependent species such as *H. amphibius*, *K. leche*, *Aonyx capensis* and *Phoenicopterus roseus*, plus wetland-adapted species such as *Kobus ellipsiprymnus* and *A. bondi*. One of the spring vents, which cut through Units P–N (∼280 kya) and was sealed by Unit M (157 ± 21 kya), produced a partial cranium of basal *H. sapiens* dated to 259 ± 35 kya using ESR on the only tooth (right M^3^) found in the same depositional context (Grün *et al.* 1996; Smith *et al.* 2015). The Florisbad specimen is now considered as an early form of the *H. sapiens* clade (Grün and Stringer 2023). Pollen from Unit O, which is contemporary with the human fossil, indicates cool and moist grassy conditions and is thus in agreement with the scenario of a well-watered environment that may have favoured human occupation (Scott *et al.* 2019).

**Table 1.** Classification systems of sedimentary units at Florisbad.

Toffolo *et al.* (2017)	Kuman *et al.* (1999)	Archaeological association
1	-	Late LSA and potsherds
2	-	LSA Lockshoek Industry
3	-	-
4	B, C, D	LSA Lockshoek Industry (in B)
5	E	MSA
6	F	MSA
7	G, H, I, J, K, L	MSA
8	M, N	MSA
9	O	Early MSA
10	P	Early MSA
11	Q	-

The recent study by Pinder (2021), based on multiple grain thermally transferred OSL (TT-OSL) of quartz and post-infrared infrared stimulated luminescence (post-IR IRSL_250_) of feldspar, produced younger ages for the same trench sampled by Grün *et al.* (1996) (the ‘Dreyer section’ in the main excavation) (Figures 5 and 6), although samples were not always collected at exactly the same depth within a given sedimentary unit. Results indicate that Units P–N present an age of ∼190 kya, with the inverted age of Unit O in between at 136 ± 7.9 kya (TT-OSL) and 158 ± 7.5 kya (post-IR IRSL_250_). On the contrary, the age of Unit M of 149 ± 13.1 kya overlaps with the age obtained in the earlier study. These new ages from the sedimentary units cut by the spring vent where the cranium was found would make the ESR age of the human tooth inconsistent with the stratigraphy, i.e. too old. However, the TT-OSL determination obtained from Pit 3, located 60 m to the north of the Dreyer section, provided an age of 254 ± 21.4 kya for the base of Unit 10 (equivalent to Unit P), immediately above bedrock, which is indeed consistent with the results of Grün *et al.* (1996) for Unit P. Unit 8 (equivalent to Units M–N) produced three ages spanning ∼213–187 kya, whereas Unit 9 (equivalent to Unit O) yielded a slightly inverted age at 176 ± 11.6 kya (TT-OSL), which nevertheless overlaps with the range of the unit above. The discrepancies between the two dating studies lie mainly in the ages of Units O and 9 (‘Peat I’), which according to Pinder (2021) might be underestimated due to a possible error in the determination of the water content used in the calculation of the dose rate. Using a revised water content of 50 ± 5%, the TT-OSL age of Unit 9 would be 204 ± 13.2 kya, which overlaps with the age of Unit O of 281 ± 73 kya, affected by a large standard deviation caused by saturation of the quartz OSL signal, and differs slightly from the ESR age of the human tooth (259 ± 35 kya) at 1σ. In addition, the TT-OSL age of Unit P in Pinder (2021), 185 ± 9.6 kya, was obtained from a sample collected close to the boundary with overlying Unit O, and not close to bedrock as in the earlier study, which could explain the discrepancy. Considering the combined results of Pit 3 and the Dreyer section, and the issues related with the estimation of past water content, the study by Pinder (2021) appears to support the results of Grün *et al.* (1996) and an age of the human fossil within MIS 7 or late MIS 8.

The absence of stable land surfaces in the peat and lacustrine layers explains the paucity of artefacts recovered from the basal units, a total of just 112 artefacts combining the 1950s and 1980s excavations (Kuman *et al.* 1999). During the 1950s excavations, Units P–N produced artefacts consistent with the early MSA technocomplex, whereas the 1980s excavations recovered a few MSA artefacts of poor diagnostic value from the same units (Meiring 1956; Kuman 1989; Kuman *et al.* 1999). The early MSA is currently ill defined in South Africa due to the scarcity of well-dated assemblages and its rather regional character, but the general consensus is that it is older than ∼130 kya (Lombard *et al.* 2022). At Florisbad, the early MSA includes larger cores compared to later assemblages, mainly multiple-platform cores, and a large proportion of side-struck flakes, as well as a large variety of raw materials including different types of igneous rocks, whereas later MSA levels are dominated by hornfels. The artefacts unearthed in Units M-H during the 1950s (N* *= >143) and 1980s (N* *= 368) are clearly MSA and include mainly side-scrapers (Sampson 1972, 1974; Kuman 1989; Kuman *et al.* 1999), although they are not attributed to a specific technocomplex *sensu* Lombard *et al.* (2022).

#### Erfkroon and Baden-Baden

The other two sedimentary deposits dated to the second half of the Middle Pleistocene were found at Erfkroon, a large donga on the right bank of the Modder River 66 km northwest of Bloemfontein, and at Baden-Baden, a spring site 12 km northeast of Dealesville. At Erfkroon, the lowermost allostratigraphic unit within the Orangia Terrace includes the Lower Gravels and Green Sands (from bottom to top), which represent a channel facies deposited under high-energy conditions. These sedimentary units produced OSL, IRSL and ESR (early uptake) ages in the range ∼183–113 kya, thus covering the end of the Middle Pleistocene and the onset of the Upper Pleistocene, and include Florisian fauna and rolled early MSA artefacts (Churchill *et al.* 2000; Tooth *et al.* 2013; Brink *et al.* 2016; Bousman *et al.* 2023a). At Baden-Baden, which features only a Later Stone Age (LSA) occupation dated to the Holocene, the bottom portion of a 5-m auger collected at the base of a sand dune next to the spring produced an OSL age of 164 ± 15 kya, indicating that the basal sediments, currently unexcavated, might well be older (van Aardt *et al.* 2016: Figure 2B). In addition, the dated sediments are consistent with deposition under waterlogged conditions typical of springs and pans, possibly during a wet phase (van Aardt *et al.* 2016: Table 1).

### Upper Pleistocene

#### Florisbad, Vlakkraal and Baden-Baden

The onset of the Upper Pleistocene (0.129–0.012 mya) is marked by the Last Interglacial period during Marine Isotope Stage 5. This period is very well represented at Florisbad, where intact occupation surfaces were exposed in the 1980s excavations immediately above the contact between Unit G and Unit F of Kuman *et al.* (1999; Units 7 and 6, respectively, in Toffolo *et al.* 2017), dated by ESR to 121 ± 6 kya (Grün *et al.* 1996) and to the range ∼170–111 kya by luminescence (Pinder 2021) (Figure 5 and Figure 6). Unit G is a peat layer on top of which a beach facies (Unit F) accumulated as a result of wind and wave action linked to the nearby Soutpan palaeolake. Pollen analysis indicates that the bottom portion of Unit G exhibits an increase in grasses, Cyperaceae and Asteraceae, whereas Amaranthaceae increase towards the top; Unit F is rich in grasses and Cyperaceae, representing local moisture availability and swampy conditions (Scott *et al.* 2019). A large number of MSA hornfels artefacts (N* *= 1654) and fossils (N* *= 573), including specimens in anatomical articulation, as well as an intact hearth, were found within a thickness of 45 cm in the lower portion of Unit F, indicating that human occupations took place on the lake shore and were gradually buried by windblown sand reworked by low-energy water (Kuman *et al.* 1999; Toffolo *et al.* 2017). Based on the large proportion of small flaking debris and the few formal tools consistent with a cutting function represented in the assemblage, the latter can be regarded as the result of expedient flaking during multiple short visits to the site, most likely related to hunting activities (Kuman 1989; Kuman *et al.* 1999). This interpretation is supported by the predominance of bovid species under 100 kg and the presence of cut marks on some bones, as well as evidence of burning and marrow extraction, although some hippopotamus remains in articulation and representative of different body parts indicate an opportunistic scavenging component. In particular, the human groups that lived at the site selected specific species such as *Damaliscus dorcas*, *D. niro*, *A. bondi* and *K. leche*, and almost always young adults and prime-aged animals, indicating highly focused hunting strategies (Brink 1987, 1988). This scenario was further corroborated by the 1990s excavations, which expanded the artefact and bone assemblages from Unit F and identified activity areas related to carcass processing and consumption, based on the spatial distribution pattern of *Hippopotamus* and *Damaliscus* bones (Brink and Henderson 2001; Henderson 2001). After MIS 5e, the only other MSA occupation at Florisbad was unearthed in Unit E, which is a thin (∼20 cm) layer of peat excavated in the 1980s that produced an assemblage of 116 artefacts in poor preservation conditions; it is possibly dated to MIS 5, based on recent luminescence ages of Unit F (111 ± 9.6 kya) and Unit D (12.1 ± 1.6 kya) obtained from samples collected close to the boundaries with Unit E (Kuman *et al.* 1999; Pinder 2021). Unit E exhibits large proportions of Amaranthaceae and Asteraceae pollens, the latter perhaps indicating karroid conditions (Scott *et al.* 2019). The recent dating programme by Pinder (2021) also demonstrated that all the sediments in Units B–D in the Dreyer section and Units 1–4 in Pit 3 are of Holocene age, thus confirming previous research by Scott and Nyakale (2002) based on radiocarbon dating of peat and showing the existence of an extensive sedimentary gap spanning almost the entirety of the Upper Pleistocene.

South of Florisbad, the Vlakkraal spring produced MSA artefacts and Florisian fossils consistent with an Upper Pleistocene age, but the site remains undated and the relocation of the original excavation areas is not possible due to the lack of accurate records (Wells *et al.* 1942). At Baden-Baden, a sediment auger from the spring mound yielded an OSL age of 113 ± 13 kya for sediments deposited under waterlogged conditions similar to the aforementioned Middle Pleistocene deposits from the dune auger (van Aardt *et al.* 2016: Figure 2B and Table 1). The sediment sample from the spring mound is rich in saddle short cell phytoliths typical of C_4_ grasses (chloridoids and aristidoids) that thrive in warm and moderately wet to arid environments. Both augers produced additional OSL ages of 38 ± 3 and 35 ± 3 kya, respectively, from sandy sediments accumulated through wind deposition that might predate a wetter period, as suggested by the phytolith assemblages extracted from these two samples, which are characterised by the predominance of short cells of C_3_ grasses, indicating regionally cooler and wetter conditions. Trench 3, excavated closer to the wetland next to the mound, delivered an OSL age of 77 ± 6 kya for spring sediments, consistent with wetter conditions during MIS 4 (van Aardt *et al.* 2016: Figure 2B and Table 4). In addition, the same trench included pollen types at 26 ± 1 kya typical of fynbos elements that are probably associated with cooler, grassy conditions (van Aardt *et al.* 2016).

#### Erfkroon

Several MSA and LSA occupations and Florisian fossil localities dated to the Upper Pleistocene were unearthed at Erfkroon within the Lower Grey and Upper Grey beds in the Orangia Terrace. Area Lower F produced Levallois flakes and blades made of hornfels and dated by OSL between ∼99 and ∼82 kya, although it is not clear whether they are in primary deposition (Morris 2019; Bousman *et al.* 2023a). Area B yielded an assemblage rich in flakes and blades in primary deposition, dated to ∼55 kya by OSL. Based on phytolith analysis, mesic C_3_ conditions possibly linked with wetlands prevailed in this period, although samples were collected from the ‘dating profile’, located on the opposite side of the donga drainage. Data from carbon stable isotopes in sediments support the occurrence of C_3_ grasslands in the same period. Additional, but smaller MSA artefacts were found in secondary deposition in Area C, dated to ∼39–38 kya. Early LSA assemblages were excavated in Areas Upper F, M and O and are dated to ∼20–19 kya in Upper F. Robberg occupations were found in Areas K and L and are currently the only such occurrence in the western Free State (Palmison 2014). Fossil teeth found in Area L, but not associated with the artefacts, were dated by ESR to ∼34–18 kya. Among the Florisian faunal remains, notable specimens are a complete *M. priscus* horncore with attached cranial fragments and the articulated front half of an *E. capensis* (Lyons *et al.* 2014; Brink *et al.* 2016; Bousman *et al.* 2023a).

#### Lovedale

**Figure 7.**
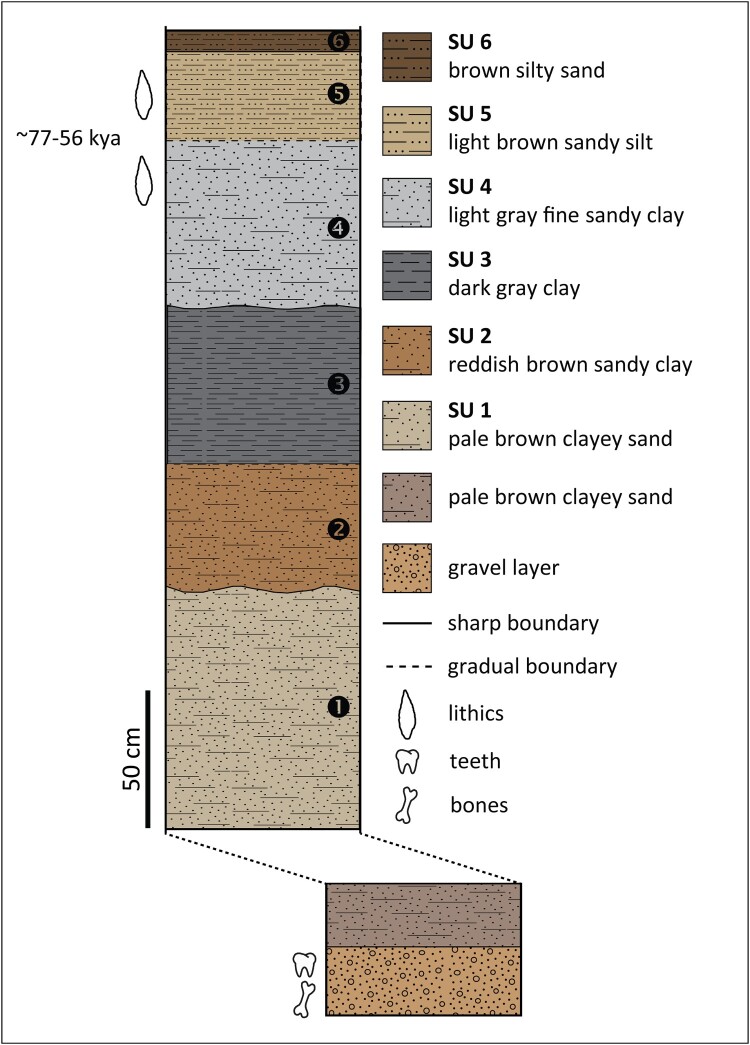
Stratigraphic section of Lovedale, showing the location of artefacts and fossils, modified from Richard et al. (2023) (SU: sedimentary unit).

About 11 km upstream of Erfkroon, Lovedale is a small donga located on the western side of a low dolerite kopje on the left bank of the Modder River. It produced an *in situ* MSA occupation dated to ∼77–56 kya by OSL within a sedimentary sequence that accumulated during the latter portion of MIS 5 and MIS 4 (Figure 7). The artefact assemblage (N = 794), composed almost entirely of hornfels, is dominated by unifacial points of two types, triangular and ‘Lovedale’, which lend support to the interpretation that the site was a hunting preparation station along the river. The former are typical MSA triangular points with facetted platforms, whereas the latter exhibit trimmed tips and bifacial trimming of the base aimed at the removal of the striking platform and bulb of percussion, presumably for hafting purposes, thus indicating the existence of two distinct production strategies (Wroth *et al.* 2022). The ‘Lovedale’ type, which appeared at Lovedale at ∼77–69 kya, finds a few parallels in the Free State, especially at Rose Cottage Cave (layer LEN) (Harper 1997), and raises the question of its relation to the coeval Still Bay technocomplex along the coast of South Africa, characterised by fully bifacial points (Lombard *et al.* 2022). A small faunal assemblage, found in a gravel layer not stratigraphically connected with the excavation area, includes the teeth of small-sized bovids, possibly *A. bondi*, as well as bones of Alcelaphini and a partial mandible of *K. leche*, a water-dependent species reflecting the wetland component of the river (Wroth *et al.* 2022). This material was dated by ESR coupled with uranium-series dating (ESR/U-series) to 64 kya, which is a weighted mean age used as minimum age for the gravel layer due to uranium leaching in the enamel (Richard *et al.* 2022, 2023). Additional environmental information was extracted from the phytolith assemblage of the occupation layer, which is characterised by a large proportion of chloridoid short cells typical of C_4_ grasses adapted to arid environments, indicating locally dry conditions at the beginning of MIS 4 (Wroth *et al.* 2022). Interestingly, Lovedale is currently the only MSA site along the Modder dated to this period, which underlines the importance of freshwater in periods of climate stress.

#### Kranskraal, Mitasrust and Waterval

About 25 km northeast of Bloemfontein, in the mid-upper reach of the Modder, three dongas have yielded significant quantities of Upper Pleistocene artefacts and fossils (Figure 3). Kranskraal, near Maselspoort, was excavated in the 1920s by van Hoepen (1932b), who unearthed fossils and MSA artefacts similar to the assemblage from Lovedale. However, the site has never been dated by absolute methods and for that reason it is impossible to place it in the chronological sequence of the region. The same applies to Mitasrust, a large donga 8 km downstream of Kranskraal, where Rossouw (2006) collected Florisian fossils and MSA and LSA artefacts of unknown age, and to Waterval, a donga 10 km upstream of Kranskraal, where Trower (2010) collected Florisian fossils and MSA and LSA artefacts.

#### Other river catchments

Sporadic finds consistent with an Upper Pleistocene age have been reported as a result of survey programmes focused on specific portions of other river catchments in the Free State. Within the framework of the Orange River Scheme during the 1960s, C.G. Sampson identified several sites in the basins of the Vanderkloof and Gariep Dams. Some of the localities were excavated, most notably the site of Orangia (Figure 2), which produced MSA artefacts and eight semi-circular structures made of dolerite cobbles possibly representing windbreaks and sleeping hollows (Sampson 1968). Unfortunately, none of the sites was dated by absolute methods and the flooding of the basins prevents further research in that direction. Berger and Brink (1996) identified a human patella in a fossil assemblage collected in 1957 by J. Kitching in a donga on the southern bank of the Riet River south of Reddersburg (Figure 2), which prompted the collection of more fossils and artefacts from the site. The fossils are of Florisian age and include *K. leche*, indicating one of its southernmost occurrences, whereas the artefacts show features consistent with MSA technology. The site was not dated and based on the finds its age could fall anywhere in the late Middle and Upper Pleistocene. At the Alexandersfontein pan south of Kimberley, in the Northern Cape portion of the Riet basin, final MSA artefacts were found in MIS 3–2 lake deposits related to a high lake stand of the pan (Butzer *et al.* 1973; Carr *et al.* 2023). Surveys along the Vet, Doring and Sand Rivers south of Welkom produced fossil and archaeological localities rich in Florisian material and MSA and LSA artefacts, although they are not dated by absolute methods (Brink *et al.* 1999; de Ruiter *et al.* 2011). Finally, excavations at the Sunnyside 1 donga on the southern bank of the Little Caledon River south of Clarens in the eastern Free State unearthed a final MSA or transitional MSA/LSA occupation dated to 30.5 ± 1.4 kya by OSL within a stratigraphic sequence that spans at least the last 65,000 years. Phytolith analysis showed that the earlier portion of the sequence is characterised by C_3_ mountain grassland indicative of moist local conditions within a cool climate at the regional scale, whereas at the time when the artefact layer accumulated phytoliths suggest a temperate C_3_ grassland environment (Kent and Scholtz 2003; Henderson *et al.* 2006).

#### Rose Cottage Cave

**Figure 8.**
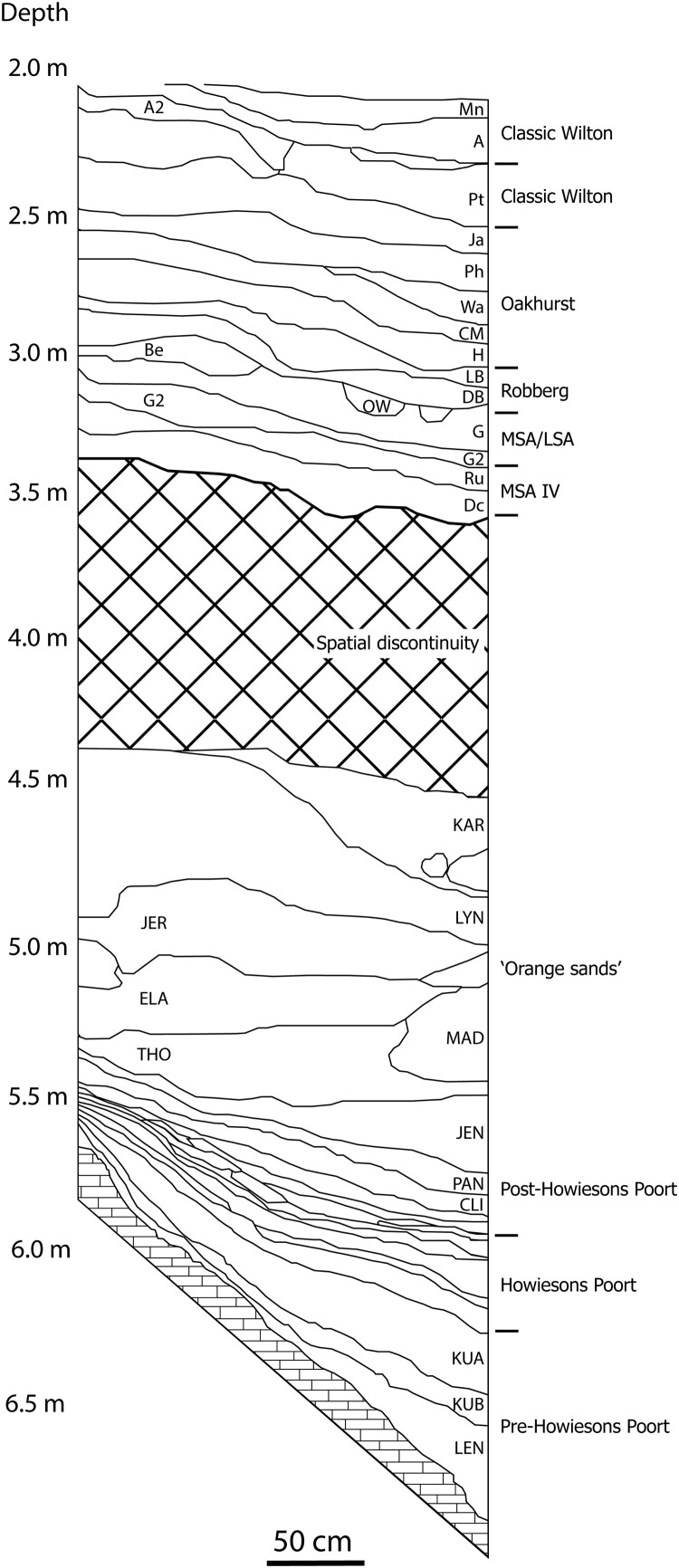
Stratigraphic section of the MSA and LSA layers at Rose Cottage Cave, modified after Pienaar *et al.* (2008).

Rose Cottage Cave, located near Ladybrand close to the Free State’s border with Lesotho (Figure 2), is the only MSA rock-shelter site in the Free State. It was excavated between 1943 and 1946 by B.D. Malan, in 1962 by P. Beaumont and between 1987 and 1997 by L. Wadley and P. Harper (Wadley and Harper 1989; Harper 1997; Wadley 1997). Extensive dating programmes based on OSL, thermoluminescence and radiocarbon show that the site was occupied discontinuously during the last ∼96,000 years (Valladas *et al.* 2005; Pienaar *et al.* 2008; Loftus *et al.* 2019) (Figure 8). The basal layer (LEN) features a pre-Howiesons Poort component (∼96–74 kya) characterised by unifacial points exhibiting trimming of the bulb of percussion similar in technology and age to the ‘Lovedale’ points discussed above (Harper 1997). The Howiesons Poort assemblage (∼67–54 kya) is one of the very few found in the interior of South Africa and the only one found within the Free State; it is followed by a thick sequence of deposits bearing post-Howiesons Poort artefacts (∼64–35 kya) (Valladas *et al.* 2005; Soriano *et al.* 2007; Pienaar *et al.* 2008). A final MSA assemblage is dated to ∼35–30 kcal BP. After a gap between 30–25 kcal BP, the Pleistocene sequence is concluded by the early LSA layers dated to ∼30 kcal BP and a Robberg occupation dated to ∼16 kcal BP (Loftus *et al.* 2019). Due to diagenesis, no faunal remains are preserved in the MSA layers. The only available palaeoenvironmental proxy is charcoal, analysis of which revealed that deciduous woodland was prevalent during MIS 5, whereas evergreen woodland became more common in MIS 4 and 3, suggesting drier conditions in the grasslands of the western Free State at this time (Lennox and Wadley 2022).

## Discussion

What emerges from the studies described above is that our knowledge of environmental change and human presence in the Free State during the Pleistocene is quite fragmentary and represented by a few sites scattered on a significantly large surface area extending over ∼130,000 km^2^. At this stage, we cannot link sub-continental climate systems to local environmental variability and human occupation with much confidence due to the lack of long and continuous palaeoenvironmental records associated with well-dated artefacts, with the exception of the latter portion of the Pleistocene (e.g. Scott *et al.* 2022).

With regard to the Lower Pleistocene and the first half of the Middle Pleistocene, the evidence is simply too scant to draw any conclusion about human settlement dynamics in the Free State. While the large numbers of Acheulean handaxes recovered along the Vaal may be considered proportional to the density of human occupation, it is also true that many occurrences were found in secondary deposition in river gravels. Sporadic surface finds of Acheulean artefacts do not bear any chronological depth although they may inform on spatial patterning related to hornfels quarries and water sources, as it was observed in the Karoo (Sampson 1985), assuming no post-depositional displacement of the artefacts took place. The faunal record of Cornelia-Uitzoek provided major insights into the evolution of the Free State grasslands towards more arid conditions and their biogeographic links with eastern Africa. However, the available information about Lower and Middle Pleistocene climate systems is largely based on environmental proxies extracted from ocean cores (e.g. Caley *et al.* 2018).

Localities featuring Fauresmith artefacts are more common in the landscape of the Free State but are mainly confined to surface scatters, in some cases in secondary deposition (river gravels), with the only possible exceptions being Brakfontein and Pniel 6. While the absence of sites with stratified fossils and sediments in primary deposition hampers the establishment of palaeoenvironmental and chronological sequences, the presence of many surface lithic assemblages may suggest a more widespread human occupation during the second half of the Middle Pleistocene. Given that the Fauresmith technocomplex overlaps in time with the appearance of *H. sapiens* in Africa, and particularly the Free State (Florisbad cranium), there is much scope in better understanding the tempo and environmental context of this archaeological period, as well as the following early MSA technocomplex (Herries 2011; Underhill 2011).

The second half of the Middle Pleistocene, roughly from Marine Isotope Stage (MIS) 8 until the end of MIS 6, is the first period that features substantial archaeological and palaeoenvironmental data, represented by the basal sedimentary deposits at Florisbad and their contents, namely the *H. sapiens* cranium, early MSA artefacts, faunal remains, pollen and enamel stable isotopes from teeth recovered in spring vents. These different lines of evidence contributed to the reconstruction of the Florisian grassland ecosystem during the Middle Pleistocene, which was drier and more open compared to Cornelian landscapes, yet much better watered compared to the Upper Pleistocene and Holocene, as indicated by the contemporary occurrence of wetland species such as *A. bondi* and *K. leche*. These marker species are found in Florisian contexts ranging from the Okavango wetlands of northern Botswana to Cradock in South Africa’s Eastern Cape Province, suggesting that an extensive system of palaeolakes existed in the interior of southern Africa at different stages during the Middle and Upper Pleistocene. This is an anomaly within the long-term trend towards aridification that started in the Cenozoic, which might have offered refugia for animal populations and human groups. Part of this system of palaeolakes is today represented by the panfield of the western Free State (Brink 2016). In addition, evidence from pollens shows that the human fossil and the most intense period of occupation during MIS 5e are linked to increased moisture availability at a local level (Scott *et al.* 2019). Despite this wealth of information, Florisbad remains an isolated case, although recent research at Baden-Baden (dune auger) and Erfkroon (Lower Gravels and Green Sands) indicates that similarly aged sedimentary deposits representing increased freshwater availability might be preserved elsewhere in the province.

The Upper Pleistocene is certainly the period characterised by the highest density of archaeological and palaeontological sites, which are found in several river catchments and at springs. Geochronological data from Florisbad, Baden-Baden, Erfkroon, Lovedale, Sunnyside 1 and Rose Cottage Cave afford some degree of robustness to the narrative of changing environments in the Modder basin and the foot of the Maloti-Drakensberg. At Florisbad, the MIS 5e occupation surfaces offer the best glimpse of foraging strategies and use of space in the open landscape (besides the undated sleeping hollows at Orangia), and underline the focal role of the spring and the nearby Soutpan palaeolake for human groups in the open grassland environment. During MIS 5a, the presence of lithic points of same age and similar technology at Lovedale and Rose Cottage Cave, including possible parallels in between at Vlakkraal (Wells *et al.* 1942: Figure 2) and Kranskraal (van Hoepen 1932b: Plate VI), points toward a certain degree of technologic coalescence in the region, perhaps reflecting a cultural tradition as observed at coastal sites (Wroth *et al.* 2022). In general, MIS 5 and 4 appear to have been periods of greater water availability based on the accumulation of overbank deposits at Erfkroon and Lovedale and the occurrence of sedimentary features consistent with submerged contexts at Florisbad and Baden-Baden. A similar scenario emerges based on data extracted from sediments at Kathu Pan (Lukich *et al.* 2020) and speleothems from Ga-Mohana Hill North (Wilkins *et al.* 2021), ∼290 km to the northwest in the Northern Cape Province; phytoliths from the basal levels at Melikane in eastern Lesotho lend support to this interpretation (Stewart and Mitchell 2018). The evidence from phytoliths in the Free State is less continuous and likely reflects more local conditions, as suggested by the dry spells identified within the MIS 5a/4 occupation at Lovedale and in different MIS 4–3 contexts at Erfkroon, whereas Sunnyside 1 experienced a moist environment during MIS 4.

Recent research has shown that the Last Glacial Maximum (LGM) in the interior of southern Africa was a colder but wetter period compared with today (Scott and Neumann 2018; Engelbrecht *et al.* 2019; Scott *et al.* 2022; Carr *et al.* 2023). Pollen sequences from Elim (Scott *et al.* 2013) and Braamhoek (Norström *et al.* 2014) in the eastern Free State, as well as from Baden-Baden in the west (van Aardt *et al.* 2016), include the *Stoebe* type indicative of cooler conditions during MIS 2. More local proxies, such as the phytolith assemblages at Erfkroon, seem to indicate drier conditions during MIS 2. Similarly, the faunal record of Damvlei, a terminal Pleistocene/Holocene site located 1.5 km upstream of Lovedale with sediments dated by OSL to 27 ± 3 kya (maximum age), shows that the Florisian assemblage is notably devoid of water-dependent species (Toffolo *et al.* 2023). However, the chronological control at these sites is not accurate enough for the LGM. To the east, radiocarbon dating of the early LSA and Robberg layers at Rose Cottage Cave indicates that the site was probably unoccupied during the LGM. To the west, the absence of Robberg assemblages at open-air sites is noteworthy, except for Erfkroon (Palmison 2014). Besides the limited absolute dating available and the little LSA research carried out at open-air sites, the discrepancies between sub-continental climate models and part of the Free State evidence for MIS 2 may be due to the different spatiotemporal scales of the palaeoenvironmental proxies considered.

### Future directions

While vast swathes of the Free State are indeed understudied, if not completely unknown (e.g. the seasonal tributaries of major rivers, springs, many pan lunettes) due to the lack of systematic survey programmes, other factors have contributed to the current situation. First, most of the sites excavated before the 1990s could not be dated by absolute methods because trapped-charge dating was not routinely applied and radiocarbon could provide ages only for the very last portion of the Pleistocene, in the few cases where organics such as charcoal and bone collagen were preserved (something that in any case is usually not true of open-air sites). This obviously hampered a proper age assessment of lithic/faunal assemblages and palaeoenvironmental proxies, most notably at well-preserved sites such as Brakfontein, Orangia, Vlakkraal and Kranskraal, some of which are now lost. Second, the vast majority of sites are located in the open landscape and thus within active sedimentary systems that are prone to erosion and bioturbation. This is particularly apparent at Florisbad, where the Permian/Jurassic bedrock is covered by sediments that are roughly 300,000 years old, which must have been deposited after earlier sediments were eroded away (Grün *et al.* 1996; Pinder 2021). For this reason, it is difficult to find sediment pockets dating to the Lower and Middle Pleistocene, as at Cornelia-Uitzoek.

Despite the limitations imposed by the local geology and geomorphology, one can think of possible ways to identify more promising portions of the territory that could be selected for survey. With regard to alluvial terraces, it would seem that riverbeds bordered by dolerite kopjes are more likely to preserve older sediments because these hard geologic barriers prevent the river from eroding the banks located downstream of the kopjes (King 1963). At Lovedale, for instance, the site is located immediately downstream of a kopje that borders the left bank of the Modder over 1 km. The upper 5 m of deposits span the last ∼80,000 years, although the actual depth of the sequence down to bedrock is unknown (Richard *et al.* 2022, 2023; Wroth *et al.* 2022). On the other side of the kopje, Damvlei features sediments ∼30,000 years old that rest directly on bedrock (Toffolo *et al.* 2023). The riverbed of the Modder is constrained by very few kopjes, and indeed sediments older than 200,000 years and Fauresmith and early MSA artefacts are rarely found in its dongas, as described above (Bousman *et al.* 2023a). Looking to the south, the Riet appears to be a better candidate for the preservation of Middle Pleistocene sediments due to the many kopjes located along its banks; it is not a surprise that several Fauresmith occurrences have been reported along its course (Goodwin and van Riet Lowe 1929). Beside river systems, springs might offer ideal environments for the preservation of Pleistocene sediments. If located at sufficient distance from riverbeds, they are not subject to erosion due to their inherent low-energy environment. In addition, water chemistry might favour the fossilisation of faunal remains under high pH conditions and through the incorporation of fluoride into the carbonate hydroxyapatite of bones, as demonstrated at Florisbad (Toffolo *et al.* 2015). Careful examination of the geological and geomorphological contexts might therefore improve the chances of finding older sediments and help narrow down target areas for survey.

Another major problem for the reconstruction of Pleistocene environments in the Free State is that different proxies have been used unevenly across the province, making it difficult to compare sites. Furthermore, different proxies bear different spatiotemporal scales, which must be taken into account in order to achieve meaningful interpretations of past environments (Thomas 2019; Faith *et al.* 2021; Lukich and Ecker 2022; Scott *et al.* 2022; Wroth *et al.* 2022). For instance, faunal remains are the most common find that can provide information on the evolution of grasslands and in some cases the morphological changes in certain species, such as *M. priscus*, allow the identification of specific stages within the Cornelian and Florisian LMAs (Brink 2005). However, marker species evolve over long periods of time and cannot yield high-resolution temporal data. In addition, they are not always present and some key sites may not preserve faunal remains at all. On the contrary, pollen records may produce sequences that are significantly more fine-grained at the spatiotemporal level compared to faunal assemblages, but their chances of preservation are much lower as they are usually found in peat, which is not a common sedimentary context (Scott and Neumann 2018). Phytoliths too yield refined datasets, although they are more time-averaged compared to pollen (unless they are found in short-lived contexts such as coprolites; Scott and Rossouw 2005) and they reflect more local conditions (Rossouw 2009). Similar considerations apply to carbon and oxygen stable isotopes from tooth enamel (Codron *et al.* 2008; Robinson 2023).

In order to consider the spatiotemporal scales inherent to the different palaeoenvironmental proxies, ideally one should analyse all of the latter wherever possible. This is often not the case because one or more of these proxies are not preserved due to diagenetic processes. One way to mitigate the incompleteness of the record is to extract and analyse plant biomarkers, which are often embedded in sediments and can preserve over hundreds of millennia in tropical and sub-tropical open-air environments (e.g. Magill *et al.* 2016). Compound-specific isotope analysis of molecules such as *n*-alkanes provides not only an assessment of the vegetation at a local level based on δ^13^C values, but can also inform on rainfall at a more regional level using δD values (Patalano *et al.* 2021b). Plant biomarkers thus offer the possibility of studying microhabitat variability at high temporal resolution within a larger environmental context, especially when combined with evidence from phytoliths, pollens, enamel stable isotopes, faunal assemblages and sedimentary structures (Patalano *et al.* 2021a). However, such an approach can be fully effective only in the presence of a robust chronological framework based on trapped-charge dating, which is essential for inter-site comparisons and ultimately to build regional narratives. In the long run, accurately dated sedimentary sequences of plant biomarkers associated with artefacts might enable us to link land and ocean archives based on similar proxies and spatiotemporal scales and thus place human palaeoecology in the Free State within a broader, sub-continental perspective of climate systems, aided by climate and hydrological models (e.g. Ecker *et al.* 2020; Chase 2021; Carr *et al.* 2023).

Besides large-scale survey programmes relying on a comprehensive and integrative approach to the study of archaeological and palaeontological localities, understanding Pleistocene human palaeoecology in the Free State will require a hypothesis-driven research design in order to infer causal relationships between environmental change and human evolution. In other words, one should avoid advancing explanations about relationships between ecology and human evolution based on empirical datasets and rather formulate hypotheses about such relationships that can be tested using the palaeoenvironmental and archaeological records (Faith *et al.* 2021).

In the Free State, where the Florisian wetlands and palaeolakes represented an exception in the sub-continental tendency towards aridity, the spatiotemporal distribution of freshwater in the landscape represents a major factor for human survival that can help formulating predictions about human palaeoecology (Lukich and Ecker 2022), as recently shown through hydrological modelling of pans in the Northern Cape (Carr *et al.* 2023). For instance, did freshwater availability exert selective pressure on human evolution (including cultural), ecology and dispersal in the central interior of South Africa? A trend in this sense seems to emerge based on the Middle and Upper Pleistocene evidence from Florisbad and the Modder catchment, which is worth investigating in order to explore the existence of a causal relationship between the spatiotemporal distribution of freshwater and the human settlement of the Free State. Searching sedimentary deposits along river terraces, around pan lunettes, at springs and around prominent landscape features (kopjes, vegetated sand dunes) will help test this hypothesis, although one should always keep in mind the problem of site visibility, which inevitably provides a partial view of human occupation. Fieldwork methodology will have to tackle this issue, given that most sites in the Free State grasslands, away from erosional features like dongas, are buried under a Holocene layer of windblown sand that makes them invisible (Coetzee and Brink 2003; Lyons *et al.* 2014; van Aardt *et al.* 2016; Wroth *et al.* 2022). Possible ways of mitigating the problem are to include surface scatters unrelated to water sources, at least with regard to the type of lithic technocomplex and the species represented in fossil assemblages, and to investigate erosional features and dongas where evidence of human occupation is absent in order to understand palaeoenvironments at these localities.

## Conclusions

The palaeontological record of the Free State has contributed enormously to our knowledge of the evolution of the southern African Grassland Biome during the Pleistocene, thanks to the type localities of the Cornelian and Florisian LMAs. The archaeological record of the Free State, on the other hand, is mainly confined to surface occurrences in the Lower and Middle Pleistocene (with the notable exceptions of Cornelia-Uitzoek and Florisbad) and to a number of stratified sites of Upper Pleistocene age. These are only a few points on a map, which are often difficult to link due to the lack of accurate dating and/or the fragmentary nature of palaeoenvironmental datasets. As a result, our understanding of human evolution and adaptive strategies in the grasslands of the central interior of the sub-continent is severely limited. Further fieldwork programmes including targeted survey and excavation are required in order to increase our capacity to formulate narratives about human palaeoecology in the central interior of South Africa. To achieve this goal, research will need to be based on explicit hypotheses regarding the relation between humans and their environment, and to place the spatiotemporal scale of different environmental proxies within the right perspective.
